# In severe alcohol‐related hepatitis, acute kidney injury is prevalent, associated with mortality independent of liver disease severity, and can be predicted using IL‐8 and micro‐RNAs

**DOI:** 10.1111/apt.17733

**Published:** 2023-10-02

**Authors:** Luke D. Tyson, Stephen Atkinson, Robert W. Hunter, Michael Allison, Andrew Austin, James W. Dear, Ewan Forrest, Tong Liu, Emma Lord, Steven Masson, Joao Nunes, Paul Richardson, Stephen D. Ryder, Mark Wright, Mark Thursz, Nikhil Vergis

**Affiliations:** ^1^ Department of Metabolism, Digestion and Reproduction Imperial College London London UK; ^2^ The Liver Unit St Mary's Hospital London UK; ^3^ Centre for Cardiovascular Science University of Edinburgh Edinburgh UK; ^4^ Cambridge NIHR Biomedical Research Centre Addenbrooke's Hospital Cambridge UK; ^5^ Department of Hepatology Royal Derby Hospital Derby UK; ^6^ Department of Hepatology Glasgow Royal Infirmary Glasgow UK; ^7^ University of Glasgow Glasgow UK; ^8^ Department of Hepatology Newcastle Freeman Hospital Newcastle upon Tyne UK; ^9^ Meso Scale Diagnostics Gaithersburg USA; ^10^ Department of Hepatology The Royal Liverpool University Hospital Liverpool UK; ^11^ NIHR Nottingham Biomedical Research Centre at Nottingham University Hospitals NHS Trust and the University of Nottingham Queens Medical Centre Nottingham UK; ^12^ Department of Hepatology University Hospital Southampton NHS Foundation Trust Southampton UK; ^13^ GSK Brentford UK

## Abstract

**Background:**

The prevalence, prediction and impact of acute kidney injury (AKI) in alcohol‐related hepatitis (AH) is uncertain.

**Aims:**

We aimed to determine AKI incidence; association with mortality; evaluate serum biomarkers and the modifying effects of prednisolone and pentoxifylline in the largest AH cohort to date.

**Methods:**

Participants in the Steroids or Pentoxifylline for Alcoholic Hepatitis trial with day zero (D0) creatinine available were included. AKI was defined by modified International Club of Ascites criteria; incident AKI as day 7 (D7) AKI without D0‐AKI. Survival was compared by Kaplan–Meier; mortality associations by Cox regression; associations with AKI by binary logistic regression; biomarkers by AUROC analyses.

**Results:**

D0‐AKI was present in 198/1051 (19%) participants; incident AKI developed in a further 119/571 (21%) with available data. Participants with D0‐AKI had higher 90‐day mortality than those without (32% vs. 25%, *p* = 0.008), as did participants with incident AKI compared to those without D0‐AKI or incident AKI (47% vs. 25%, *p* < 0.001). Incident AKI was associated with D90 mortality adjusted for age and discriminant function (AHR 2.15, 1.56–2.97, *p* < 0.001); D0‐AKI was not. Prednisolone therapy reduced incident AKI (AOR 0.55, 0.36–0.85, *p* = 0.007) but not mortality. D0 bilirubin and IL‐8 combined, miR‐6826‐5p, and miR‐6811‐3p predicted incident AKI (AUROCs 0.726, 0.821, 0.770, *p* < 0.01).

**Conclusions:**

Incident AKI is associated with 90‐day mortality independent of liver function. Prednisolone therapy was associated with reduced incident AKI. IL‐8 and several miRNAs are potential biomarkers to predict AKI. Novel therapies to prevent incident AKI should be evaluated in AH to reduce mortality.

## Graphical abstract

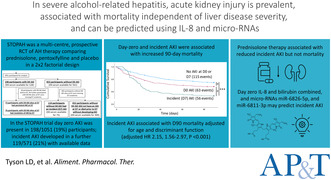

## 1 INTRODUCTION

Alcohol‐related hepatitis (AH) is the most severe manifestation of alcohol‐related liver disease (ARLD), with a 90‐day mortality of severe AH of approximately 30%.^1^, ^2^ Acute kidney injury (AKI), a reduction in renal function over hours to days, is associated with death in AH as a cause itself or associated with infection or variceal bleeding.^2^

AKI is common in severe AH, with previous literature suggesting an incidence of approximately 30%.^3^, ^4^, ^5^ However, detailed characterisation is lacking. Patients present with AKI on admission to hospital or AKI may develop during hospital stay (‘incident AKI’). For the general hospital population, incident AKI has a higher mortality rate than AKI on admission,^6^ and is preventable in 20% of cases.^7^

The relationship between AKI and mortality is well established for patients with cirrhosis.^8^ However, patients with AH follow a different disease trajectory to those with decompensated cirrhosis.^9^ In AH, AKI has been associated with mortality,^3^, ^4^ but not consistently.^5^ Further, there is conflicting evidence as to whether this relationship is independent of liver disease severity^3^ or not.^4^ Hence, therapeutic development in AH has focused on restoration of hepatocyte function, with few trials aimed at treating or preventing AKI.

The dynamic nature of AKI mandates rapid intervention to improve outcomes. However, defining AKI by serum creatinine can only report AKI hours‐to‐days after it has occurred because of the time taken for creatinine to rise after a fall in glomerular filtration rate (GFR).^10^ Clinical risk factors to identify at‐risk patients can mitigate this issue; in AH baseline systemic inflammatory response syndrome (SIRS)^3^, ^4^, ^5^ and hepatic encephalopathy may be associated with incident AKI.^4^, ^5^

Novel biomarkers have been proposed to predict AKI more accurately and at an earlier timepoint than serum creatinine and clinical risk factors.^12^ If validated, such biomarkers could facilitate prompt treatment. Putative biomarkers include serum cystatin C^14^, neutrophil gelatinase‐associated lipocalin (NGAL)^12^ and beta‐2 microglobulin (B2M)^13^; as well as proinflammatory cytokines interleukin‐6 (IL‐6),^14^, ^15^, ^16^ interleukin‐8 (IL‐8),^16^ interleukin‐18 (IL‐18),^12^ interleukin‐22 (IL‐22)^17^ and TNF‐α.^18^ Transforming Growth Factor β1 (TGF‐β1)^11^ and Transforming Growth Factor β2 (TGF‐β2)^19^ have been linked to early renal fibrogenesis, potentially acting via microRNAs (miRNA). These are short noncoding RNAs that can regulate expression by post‐transcriptional repression of specific coding RNAs (mRNA). MicroRNAs are stable in serum, can be measured by commercial assays, and hence are also attractive potential biomarkers.^20^ None of these biomarkers have been evaluated in AH.

We used samples and data from participants of the largest RCT conducted in AH to date^1^ to determine the incidence of AKI in AH and to interrogate its association with mortality. Further, we aimed to clarify clinical risk factors for AKI in AH. Finally, we screened novel biomarkers of AKI that may predict AKI at an earlier timepoint.

## 2 MATERIALS AND METHODS

### 2.1 Patient selection

All participants recruited to the multi‐centre Steroids or Pentoxifylline for Alcoholic Hepatitis (STOPAH) RCT with a measured trial baseline (day zero, D0) creatinine available were included. These patients have been described in detail previously.^1^ STOPAH utilised a 2 × 2 factorial design, with treatment groups of prednisolone, pentoxifylline, placebo and prednisolone, and pentoxifylline, with *n* = 250 in each group and a total sample size of 1092. Ethical approval was granted by the Wales Research Ethics Committee (REC 09/MRE09/59), and the work was conducted in accordance with the 2013 Declaration of Helsinki. The STOPAH trial predates the 2018 Declaration of Istanbul and does not concern organ transplantation. Written consent was obtained from each participant or from their legal representative until such time as they recovered mental capacity.

Participants in STOPAH met clinical criteria for severe AH.^1^, ^21^ Of note, patients with serum creatinine >500 μmol/L (>5.7 mg/dL) or renal replacement therapy (RRT) at screening were excluded. Outcome data were collected at day 28 (D28), day 90 (D90) and one year. The results of the STOPAH clinical trial have previously been reported: neither pentoxifylline nor prednisolone affected mortality at D90. Prednisolone reduced mortality at D28, but this did not reach statistical significance.

### 2.2 Definition of acute kidney injury

For patients with liver cirrhosis, the International Club of Ascites (ICA) definition of AKI has been widely adopted.^22^ This is a dynamic definition based on serum creatinine. This is important because serum creatinine may be normal in patients with cirrhosis and AKI because of muscle wasting, increased renal tubular creatinine secretion, and increased volume of distribution.^22^ Definitions based on a fixed creatinine cut off—traditionally 133 μmol/L (1.5 mg/dL)—may under‐report clinically significant AKI.^23^, ^24^, ^25^, ^26^ The ICA definition excludes urine output because patients with cirrhosis may be oliguric due to sodium retention yet have a normal GFR, and many patients use diuretics.^22^

STOPAH was a prospective, multi‐centre RCT. Hence, pre‐admission values for creatinine are not available. The most widely used alternative method of estimating baseline creatinine is to inversely apply the Modification of Diet in Renal Disease (MDRD) equation, as suggested by the Kidney Disease Improving Global Outcomes (KDIGO) group practice guidelines.^10^ However, this approach is inaccurate in patients with cirrhosis^27^ and is not recommended by the European Association for the Study of the Liver (EASL)^28^ or the ICA.^22^ We confirmed this by comparing formula‐based imputation methods to the lowest recorded creatinine obtained during follow‐up (participants were followed for one year), discarding results obtained during RRT (methodology described in Analysis S1; results in Figure S1).

These data demonstrate that formulae‐based imputation methods consistently overestimated baseline creatinine compared to the lowest‐recorded creatinine, consistent with the observation that patients with chronic liver disease have relatively low serum creatinine.^22^, ^27^ Estimated GFRs calculated using standard formulae are likely to significantly overestimate true GFR. Hence, although the use of the lowest‐recorded creatinine is not necessarily representative of the baseline creatinine before the development of AKI, it was the best available measure. This is summarised in Figure S2.

Taking the lowest recorded creatinine as the baseline, we used predefined criteria based on the ICA definition^22^ to classify AKI at D0 (D0‐AKI) and day seven (D7). Day 7 was the first trial visit after D0; there were no results available for the intervening period. AKI was defined as one of: an increase in serum creatinine to ≥1.5 times the participants' assumed baseline; an increase ≥26.5 μmol/L (0.3 mg/dL) above baseline; or the initiation of RRT. To avoid omitting participants admitted with AKI who died without recording a normal creatinine, participants with a creatinine of ≥133 μmol/L (1.5 mg/dL) were also defined as having an AKI, as suggested by ICA guidance.^22^ To ensure our findings were robust to alternative methods of ascribing AKI, a sensitivity analysis using traditional criteria (creatinine of ≥133 μmol/L [1.5 mg/dL] at D0 or D7) was performed for key analyses.

AKI at D7 in participants without D0‐AKI was termed ‘incident AKI’ for the purpose of identifying participants with a potentially preventable AKI using D0 clinical risk factors and novel biomarkers.

### 2.3 Outcome measures

The primary outcome measure evaluated was death at day 90 (D90), in line with current consensus guidelines and expert opinion.^29^, ^30^ Death at day 28 (D28) was evaluated as a secondary outcome measure to further illuminate the time course of the association. In the STOPAH cohort, three participants underwent liver transplantation, but all were transplanted after D90.^1^

**FIGURE 1:**
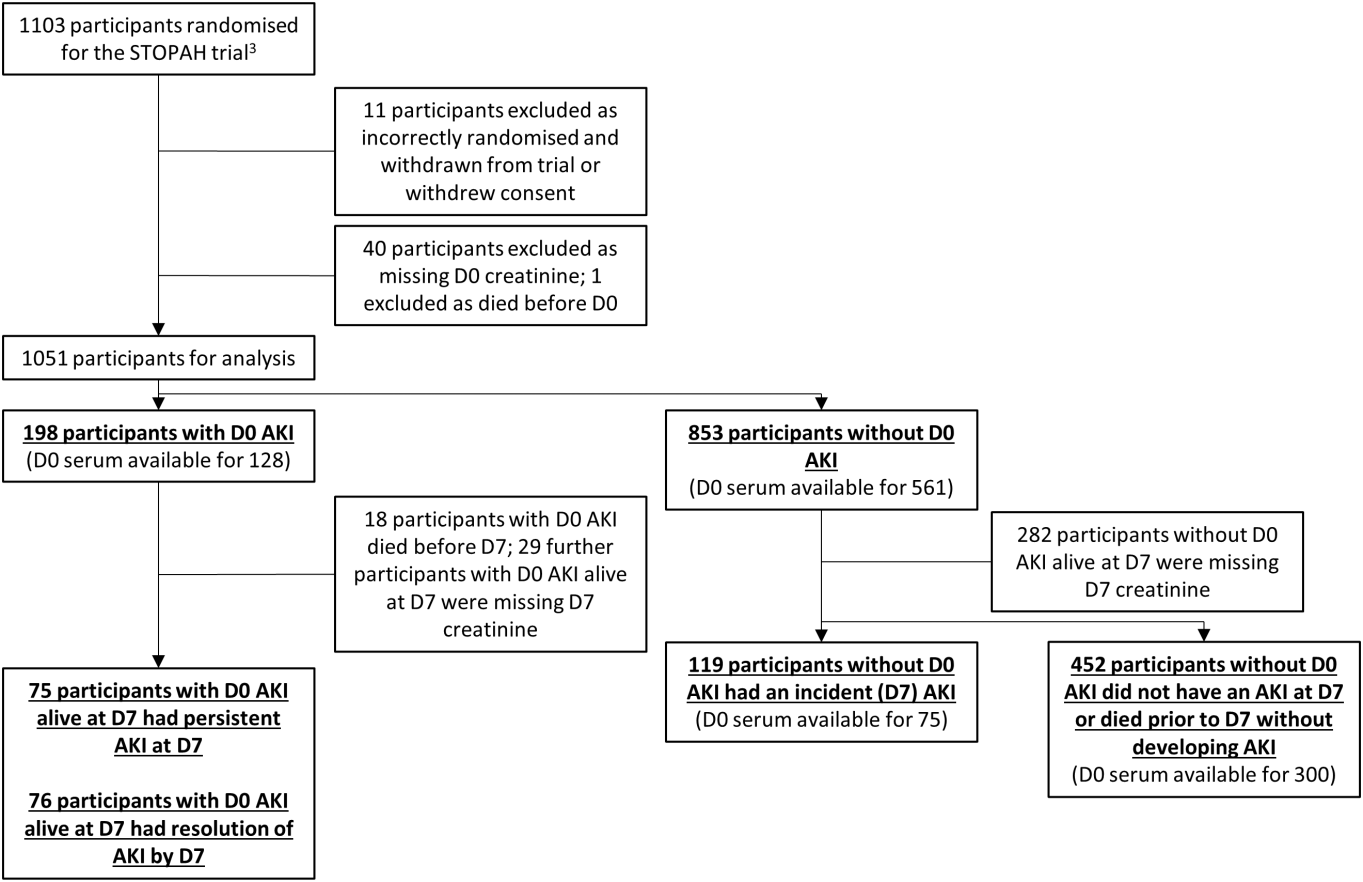
Consort diagram of novel participant classifications and availability of stored serum for biomarker analyses. AKI, acute kidney injury; D0, day zero; D7, day seven; STOPAH, STeroids Or Pentoxifylline for Alcoholic Hepatitis trial.

Two main comparisons were undertaken: firstly, participants with D0‐AKI were compared to those without D0‐AKI; secondly, participants without D0‐AKI who developed an incident (D7) AKI were compared to those without D0 or D7‐AKI. In addition, participants with D0‐AKI who had persistent AKI at D7 were compared to those whose D0‐AKI had been resolved by D7. However, this last comparison was relatively underpowered (Figure 1).

Significant associations with mortality were adjusted for age and D0‐mDF score^31^ to account for liver disease severity at D0. Maddrey's mDF was preferred to the Model for End‐Stage Liver Disease (MELD) score because the MELD score includes creatinine,^32^ which confounds the analysis due to co‐linearity. However, sensitivity analyses were performed using MELD, bilirubin, and INR to ensure significant associations were robust to different measures of liver function. The Lille score was not used as either a surrogate endpoint or a marker of disease severity since serum creatinine directly modulates the Lille model.

### 2.4 Clinical variables associated with acute kidney injury

The association of AKI with previously linked clinical variables^3^, ^4^, ^5^ and routine haematological and biochemical indices was tested at D0 and D7. Associated variables were evaluated as predictors of incident AKI.

### 2.5 Novel serum biomarkers

Stored day zero serum was available for novel analyses for 689 participants (Figure 1). Serum was collected into serum separator tubes, processed, and stored at −80°C. The concentration of putative serum biomarkers from the literature (Cystatin C, NGAL, B2M, IL‐6, IL‐8, IL‐18, IL‐22, TGF‐β1, TGF‐β2 and TNF‐α) was determined using the U‐PLEX assay platform from Meso Scale Discovery, following the manufacturers' instructions. Urine was not available.

A randomly selected subset of 39 participants (Table S1) was used for miRNA biomarker exploration. Total RNA was isolated from 100 μL of serum using the QIAGEN miRNease Serum/Plasma kit and RNA libraries were prepared using the QIAseq microRNA library kit following the manufacturer's instructions (Methods S1).

### 2.6 Statistical analysis

Survival was evaluated at D90 by Kaplan‐Meier survival analysis; significance was determined by the Breslow (Generalised Wilcoxon) test as the proportional hazard of AKI was assumed to be greater at earlier timepoints. Associations with mortality were explored by univariate and multivariate Cox regression analyses. Associations with AKI and incident infection were explored by univariate and multivariate binary logistic regression. Logistic regression was preferred to time‐to‐event analysis when testing associations with AKI because creatinine was only available for predefined study visits (D0, D7, D14, D21, D28, D90 and D365). Logistic regression was used for incident infection analysis for consistency with AKI analysis. Biomarkers were evaluated by area under the receiver operating characteristic (AUROC) analyses. Continuous parametric demographic and clinical characteristics were compared by Student's *t*‐test, non‐parametric by Mann‐Whitney U, categorical by the *χ* ^2^ test. Data were analysed in SPSS (IBM, New York). Reported P values are two‐tailed unless stated; *p* < 0.05 considered significant. Where appropriate, false discovery rate was controlled by the Benjamini‐Hochberg procedure with a false discovery rate of 5%. Missing data were addressed by pairwise deletion: participants with missing data were excluded only from those analyses where data were missing.

MicroRNA differential expression analysis was performed in R (version 4.1.3) using the edgeR package (version 3.36.0). UMI counts present at an abundance of at least 20 counts per million in 25% of libraries were normalised using the trimmed median of the M‐values method, and differential expression was determined using a 2‐group generalised log‐linear model, comparing participants with incident AKI to those without D0 or D7‐AKI. Genes were deemed differentially expressed if expression was >1.5‐fold different between the incident AKI and no‐AKI groups and the false‐discovery rate was less than 5%. Candidate miRNA biomarkers for incident AKI were selected and then evaluated by AUROC analyses. To minimise type 1 error rate, we evaluated the top five most differentially expressed miRNAs.

## 3 RESULTS

### 3.1 Prevalence of day 0 and incident acute kidney injury

Key clinical characteristics are summarised in Table 1, and the two comparator cohorts compared in Table S2. Of the 1103 participants randomised for the STOPAH trial (1092 included in the published analysis),^1^ 1051 had D0 creatinine recorded. Of these, 198/1051 (19%) had an AKI at D0 (D0‐AKI). From those without D0‐AKI, excluding those alive at D7 without D7 creatinine available, a further 119/571 (21%) developed an incident (D7) AKI (Figure 1).

**TABLE 1:** Key demographic and clinical characteristics of participants at randomisation.

	Whole cohort	D0 AKI	No D0 AKI	Incident (D7) AKI	Neither D0 nor incident AKI	Alive at D7 without D7 creatinine^a^
*N* (% of whole cohort)	1051 (100)	198 (19)	853 (81)	119 (11)	452 (43)	282 (27)
Median age, years (IQR)	49 (42–56)	50 (42–56)	49 (42–56)	49 (43–57)	49 (42–57)	48 (41–55)
Male gender, *N* (% of group)	661 (63)	124 (63)	537 (63)	77 (65)	273 (60)	187 (66)
Death by D28, *N* (% of group)	166 (16)	44 (22)	122 (14)	45 (38)	65 (14)	12 (4)
Death by D90, *N* (% of group)	273 (26)	63 (32)	210 (25)	56 (47)	115 (25)	39 (14)
Prednisolone therapy, *N* (% of group)	525 (50)	113 (57)	412 (48)	39 (33)	213 (47)	160 (57)
Pentoxifylline therapy, *N* (% of group)	527 (50)	105 (53)	422 (49)	59 (50)	216 (48)	147 (52)
Beta‐blocker use at D0, *N* (% of group)	149 (14)	27 (14)	122 (14)	22 (18)	57 (13)	43 (15)
Hepatic encephalopathy at presentation, *N* (% of group)	257 (24)	70 (35)	187 (22)	31 (26)	107 (24)	49 (17)
Gastrointestinal bleed at presentation, *N* (% of group)	79 (8)	12 (6)	67 (8)	11 (9)	34 (8)	22 (8)
Infection at presentation, *N* (% of group)	117 (11)	25 (13)	92 (11)	16 (13)	53 (12)	23 (8)
Infection at D7, *N* (% of group)	115 (11)	31 (16)	84 (10)	23 (19)	48 (11)	13 (5)
Median D0 bilirubin, μmol/L (IQR)	269 (169–415)	386 (247–520)	243 (157–387)	390 (262–470)	260 (177–385)	182 (122–305)
Median D0 INR (IQR)	1.8 (1.6–2.1)	1.8 (1.6–2.2)	1.8 (1.5–2.0)	1.8 (1.6–2.2)	1.7 (1.5–2.0)	1.8 (1.5–2.0)
Median D0 creatinine, μmol/L (IQR)	64 (53–85)	123 (91–181)	61 (51–73)	66 (52–82)	60 (50–72)	61 (51–71)
Median mDF (IQR)	55 (43–74)	63 (49–89)	54 (43–71)	64 (52–89)	53 (43–70)	52 (41–66)
Median D0 MELD (IQR)	26 (23–29)	30 (26–34)	25 (22–28)	28 (25–30)	25 (23–28)	24 (22–27)

Abbreviations: AKI, acute kidney injury; D0, day zero; D7, day seven; D28, day 28; D90, day 90; INR, international normalised ratio; IQR, interquartile range; *N*, number; mDF, Maddey's modified Discriminant Function; MELD, Model For End‐Stage Liver Disease score.

^a^ Excluded from incident AKI analyses per Figure 1.

### 3.2 Acute kidney injury, mortality and liver function

**FIGURE 2:**
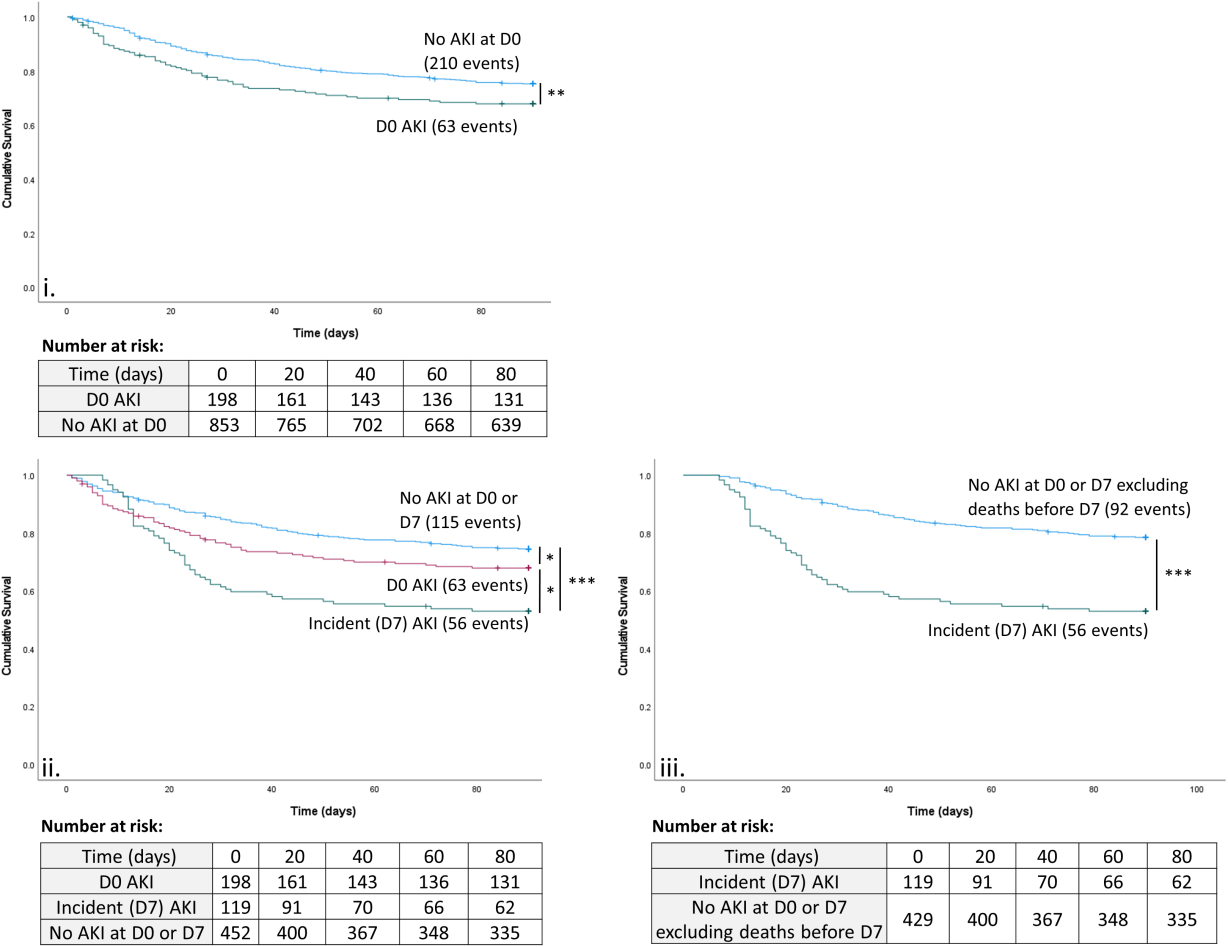
Kaplan‐Meier survival curves for participants with severe alcohol‐related hepatitis with a day zero acute kidney injury and for those without acute kidney injury at day zero (i); for day zero and incident acute kidney injury, and for those without acute kidney injury at day zero or day 7, excluding those alive at day 7 without creatinine available (ii); for incident acute kidney injury, and for those without acute kidney injury at day zero or day 7, excluding all participants without day 7 creatinine available (iii). *Beslow (Generalised Wilcoxon) *p* < 0.05; **p < 0.01; ***p < 0.001. AKI, acute kidney injury; D0, day zero; D7, day seven.

Participants with D0‐AKI had reduced 90‐day survival compared to those without D0‐AKI (Breslow *χ* ^2^ 7.00, *p* = 0.008, Figure 2i). At D90, 63/198 (32%) participants with D0‐AKI had died compared to 210/853 (25%) without D0‐AKI (Table 1; Table S2). Participants with an incident AKI had reduced survival compared to those without D0 or D7‐AKI (Breslow *χ* ^2^ 24.46, *p* < 0.001, Figure 2ii) and compared to those with D0‐AKI (Breslow *χ* ^2^ 4.99, *p* = 0.025, Figure 2ii). At D90, 56/119 participants with an incident AKI (47%) had died, compared to 115/452 (25%) participants without D0 or D7‐AKI (Table 1; Table S2). The International Club of Ascites AKI stage correlated with increasing mortality for participants with an incident AKI (Table S3).

To eliminate immortal time bias, a sensitivity analysis excluded participants who died before D7: this showed a greater difference between participants with incident AKI compared to those without D0 or D7‐AKI (Breslow *χ* ^2^ 45.49, *p* < 0.001, Figure 2iii). For participants with D0‐AKI, resolution at D7 was not associated with improved 90‐day survival compared to persistent AKI (Breslow *χ* ^2^ 0.227, *p* = 0.633).

D0‐AKI was associated with increased D90 mortality (hazard ratio 1.42, 95% confidence interval 1.07–1.88, *p* = 0.015) in Cox regression analysis; however, this was not significant when adjusted for age and D0‐mDF (adjusted hazard ratio 1.33, 1.00–1.76, *p* = 0.053). As discussed, mDF was preferred to MELD as a measure of liver function, as mDF does not include creatinine. Incident AKI was associated with increased D90 mortality (HR 2.19, 1.59–3.02, *p* < 0.001) and, in contrast to D0‐AKI, was associated independent of age and D0‐mDF (AHR 2.15, 1.56–2.97, *p* < 0.001). In sensitivity analyses, incident AKI remained associated with D90 mortality when adjusted for age and D0 MELD score, bilirubin or INR and when adjusted for age and D7 mDF, bilirubin, or INR (Analysis S1).

Both D0‐AKI and incident AKI were associated with D28 mortality, including when adjusted for age and D0‐mDF: AHR 1.58, 1.11–2.33, *p* = 0.010; AHR 2.76, 1.88–4.06, *p* < 0.001, respectively. Defining AKI as creatinine ≥133 μmol/L (1.5 mg/dL), 86 participants had a D0‐AKI and 52 participants had an incident AKI. Using this definition, both D0‐AKI and incident AKI were associated with D90 mortality, including when adjusted for age and D0‐mDF (AHR 2.49, 1.79–3.47 *p* < 0.001; AHR 3.28, 2.23–4.82, *p* < 0.001, respectively).

### 3.3 Clinical risk factors for acute kidney injury

Participants' D0‐mDF, MELD, and Glasgow Alcoholic Hepatitis Score (GAHS)^33^ strongly associated with D0‐AKI (OR 1.01, 1.00–1.01, *p* < 0.001; OR 1.29, 1.24–1.35, *p* < 0.001; OR 2.00, 1.74–2.29, *p* < 0.001, respectively) and the development of incident AKI in those without D0‐AKI (OR 1.01, 1.00–1.01, *p* = 0.008; OR 1.15, 1.09–1.22, *p* < 0.001; OR 1.41, 1.18–1.68, *p* < 0.001, respectively).

**FIGURE 3:**
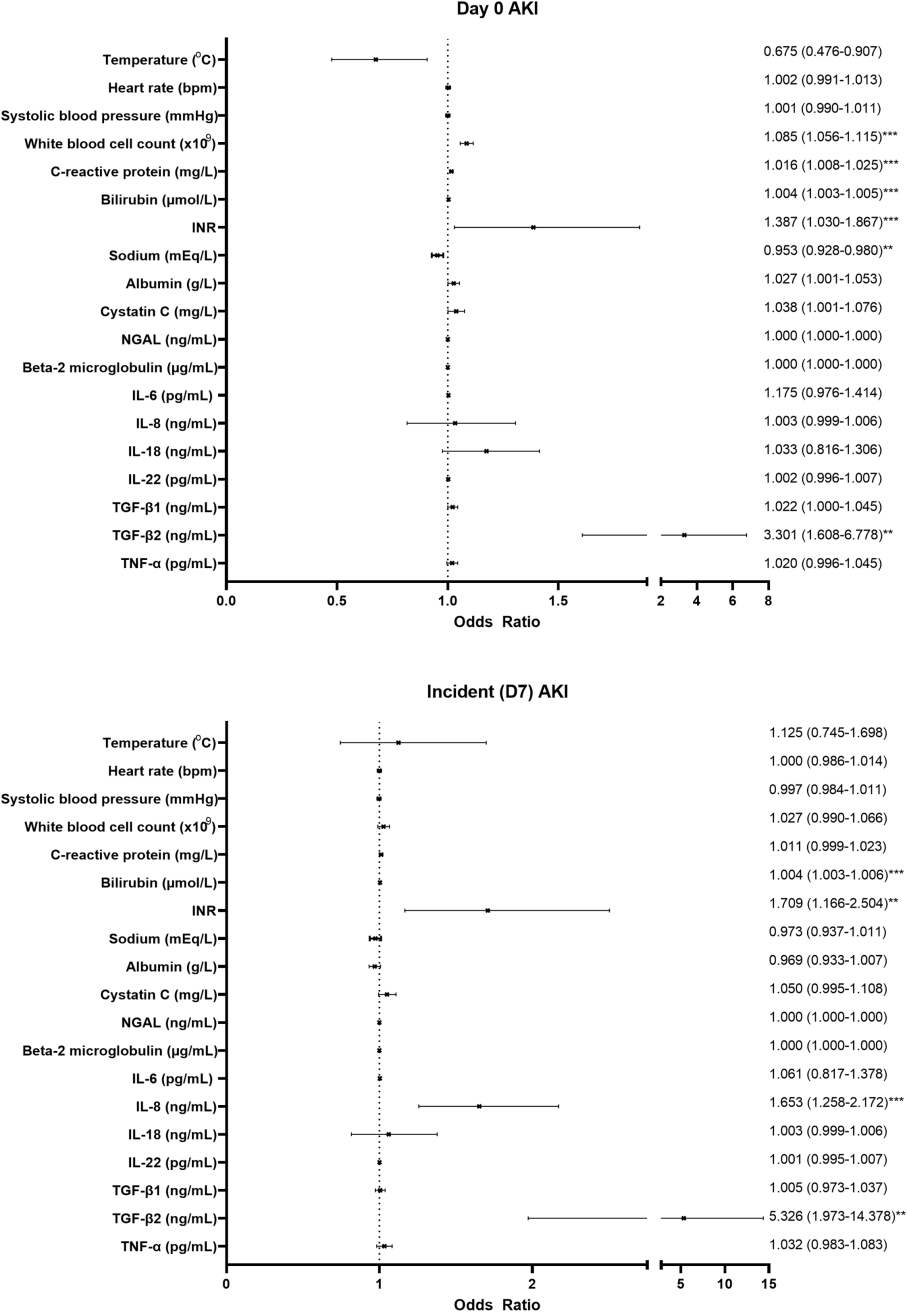
Binary logistic regression of day zero variables associated with day zero and incident acute kidney injury. *p < 0.05; **p < 0.01; ***p < 0.001 (corrected for multiple testing using Benjamini‐Hochberg False Discovery Rate 0.05). AKI, acute kidney injury; D0, day zero; D7, day seven; IL, interleukin; INR, international normalised ratio; NGAL, neutrophil gelatinase‐associated lipocalin; SIRS, systemic inflammatory response syndrome; TGF, transforming growth factor; TNF, tumour necrosis factor.

Individual clinical and laboratory variables previously associated with AKI (temperature, heart rate, systolic blood pressure at screening; white blood cell count [WBC], C‐reactive protein [CRP], bilirubin, INR, sodium and albumin) were evaluated. WBC, CRP, bilirubin, INR and sodium were associated with D0‐AKI, only D0 serum bilirubin and INR were associated with incident AKI (Figure 3).

The presence of hepatic encephalopathy at recruitment (D0) was associated with D0‐AKI, including when adjusted for the D0‐mDF score (AOR 1.92, 1.37–2.69, *p* < 0.001), but not incident AKI. Gastrointestinal bleeding and infection at recruitment were not associated with D0 or incident AKI (Analysis S1). However, incident infection at D7 was associated with incident AKI, including when adjusted for mDF (AOR 2.09, 1.21–3.61, *p* = 0.008). Incident AKI and D7 infection were both independently associated with D90 mortality (AHR 2.07, 1.50–2.86, *p* < 0.001; AHR 2.37, 1.64–3.42, *p* < 0.001, respectively).

### 3.4 Prednisolone and pentoxifylline

Prednisolone treatment was associated with reduced risk of incident AKI (OR 0.55, 0.36–0.84, *p* = 0.005), including when adjusted for the D0‐mDF (AOR 0.55, 0.36–0.85, *p* = 0.007). Prednisolone treatment was associated with reduced D28 mortality (HR 0.74, 0.54–1.00. *p* = 0.050; AHR 0.69, 0.51–0.94, *p* = 0.020) but increased mortality in the period between D28 and D90, as seen previously in STOPAH.^34^ Accordingly, no benefit was seen at D90 (HR 1.01, 0.80–1.28, *p* = 0.929).

Prednisolone treatment was not associated with developing an infection by D28 (Analysis S1) but was associated with developing an infection between D28 and D90 (AOR 1.661, 1.049–2.632, *p* = 0.031). Developing an infection by D90 associated with mortality (AHR 1.35, 1.06–1.73, *p* = 0.017).

Pentoxifylline treatment was not associated with incident AKI (OR 1.10, 0.72–1.67, *p* = 0.656) or mortality at any timepoint.

### 3.5 Beta‐blocker use

There was no association between any beta‐blocker use at randomisation and D0 or incident AKI (OR 0.95, 0.60–1.48, *p* = 0.809; OR 1.57, 0.92–2.70, *p* = 0.101, respectively). There was also no association between cardioselective beta‐blockers (atenolol, bisoprolol, metoprolol or nebivolol), non‐selective beta‐blockers (propranolol, carvedilol, or nadolol), carvedilol, or propranolol and D0 or incident AKI, including when AKI was defined as creatinine ≥133 μmol/L (Analysis S1). Beta blockers were not associated with D90 mortality.

### 3.6 Novel biomarkers for incident acute kidney injury

Day zero IL‐8 and TGF‐β2 were associated with incident AKI (Figure 3). They were evaluated as predictors of incident AKI alongside established clinical scores and laboratory indices, demonstrating weak‐moderate predictive ability (Table 2). Logistic regression analysis was used to derive potential predictive scores. A combination of D0‐bilirubin and IL‐8 (0.005 × (bilirubin, μmol/L) + 0.415 × (IL‐8, ng/mL)‐3.238) performed best (AUROC 0.726, 0.658–0.794, *p* < 0.001). For participants with both bilirubin and IL‐8 available, there was a trend to improved predictive performance with the D0‐bilirubin/IL‐8 score compared to D0‐bilirubin alone (*n* = 375, AUROC 0.726 vs. 0.701, *z* = 1.613, *p* = 0.108).

**TABLE 2:** Performance of novel biomarkers and established clinical scores and laboratory indices in predicting incident acute kidney injury at day 7 in participants without an acute kidney injury at day 0.

Day zero variable	AUROC	95% confidence interval	Two‐tailed *p* value
mDF	0.642	0.587–0.697	<0.001
MELD	0.656	0.599–0.713	<0.001
GAHS	0.612	0.554–0.670	<0.001
Serum bilirubin, μmol/L	0.674	0.619–0.729	<0.001
INR	0.587	0.529–0.645	0.004
Serum IL‐8, ng/mL	0.633	0.561–0.705	<0.001
Serum TGF‐β2, ng/mL	0.586	0.514–0.659	0.020

Abbreviations: GAHS, Glasgow Alcoholic Hepatitis Score; INR, international normalised ratio; mDF, Maddey's modified Discriminant Function; MELD, Model For End‐Stage Liver Disease score.

The differences in clinical and novel biomarkers observed between participants with and without D0‐AKI and with and without incident (D7) AKI did not vary between prednisolone and pentoxifylline treatment groups (Tables S4 and S5).

**FIGURE 4:**
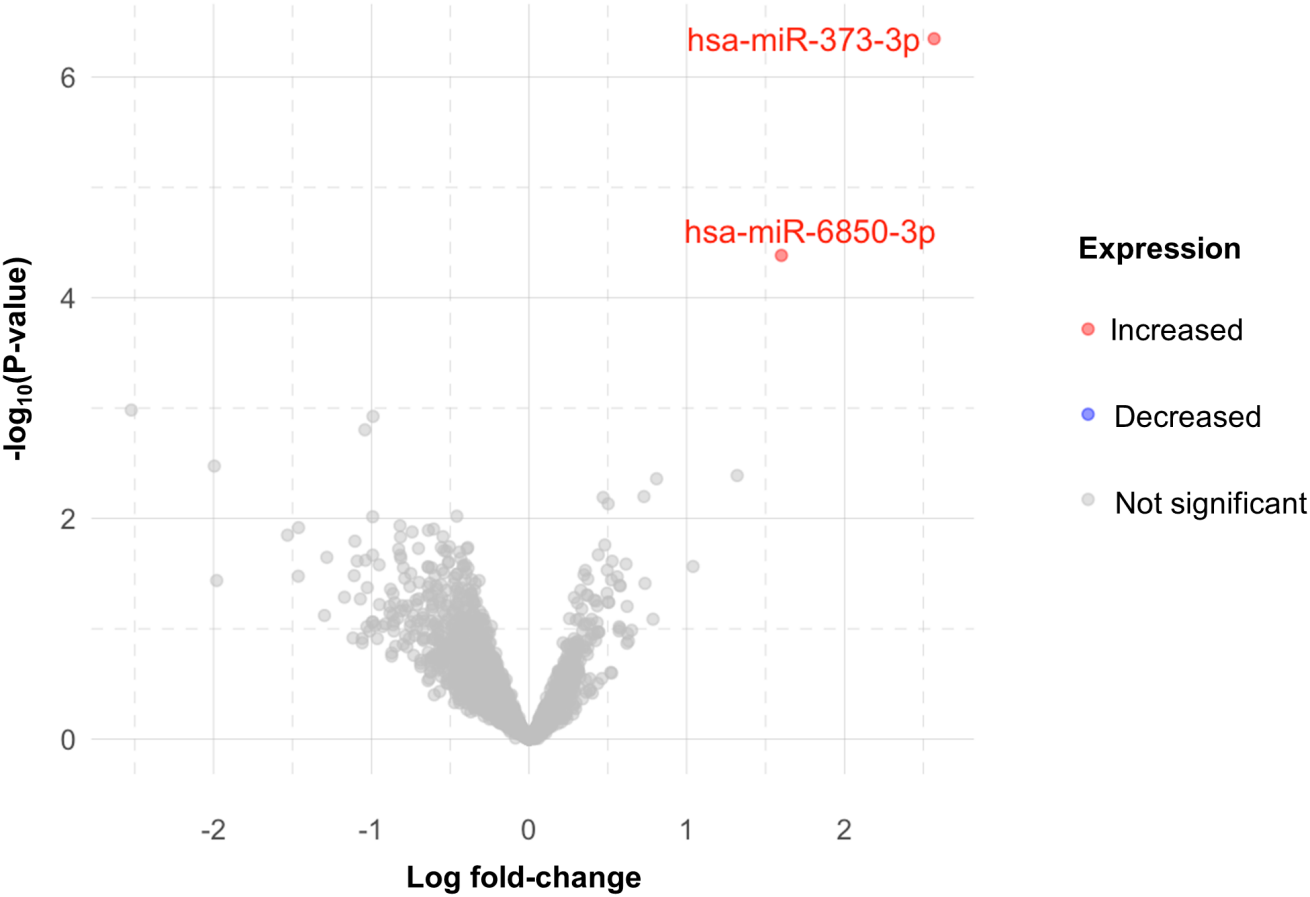
Volcano plot showing differential expression of circulating micro RNAs (measured on day zero) in participants who developed incident acute kidney injury at day seven, compared with participants who did not have an AKI at either timepoint; genes were deemed to be significantly differentially expressed if their expression was at least 1.5‐fold different between the AKI and no‐AKI groups and the false‐discovery rate was less than 5% after adjustment for multiple testing using the Benjamini‐Hochberg method.

In a differential expression analysis, two microRNAs (miR‐373‐3p and miR‐6850‐3p) were significantly upregulated at D0 in participants who developed incident AKI, compared to those without D0 or D7‐AKI (Figure 4). However, both results were due to high miRNA expression in a single patient (different for each). Of the top five most differentially expressed genes (Table S6), miR‐6825‐5p and miR‐6811‐3p looked most promising as potential biomarkers (Figure S3). Both miR‐6826‐5p (AUROC 0.821, 0.689–0.953, *p* = 0.002) and miR‐6811‐3p (AUROC 0.770, 0.625–0.915, *p* = 0.008) predicted incident AKI.

## 4 DISCUSSION

Around a fifth of participants in STOPAH presented with D0‐AKI, and a further fifth of participants developed incident AKI. Incident AKI was robustly associated with D28 and D90 mortality, independent of liver disease severity or, importantly, definition of AKI. Nearly half of participants (47%) who developed incident AKI died by D90, compared to 25% without D0 or incident AKI.

Interestingly, the association between incident AKI and mortality is independent of liver function. This suggests developing incident AKI is not merely a consequence of a severe liver injury but is an independent risk factor for mortality. Consequently, preventing incident AKI could potentially improve patient outcomes even if a treatment does not impact underlying liver dysfunction. The magnitude of the increase in mortality seen implies that kidney‐targeted therapy could result in a large survival advantage, with a potential number needed to treat as low as 4.5. This is an especially important finding given the number of failed RCTs in the field and the few treatment options available for patients.

Prednisolone treatment was associated with a reduced risk of incident AKI but without mortality benefit at D90. This suggests that the renal benefit of prednisolone are offset by the deleterious effects of corticosteroids, such as a propensity to sepsis.^34^ Glucocorticosteroids reduce renal complications following other acute illnesses and insults, for example coronavirus disease 2019,^35^ and cardiac surgery.^36^ Further work may identify the underlying mechanisms; for example, steroids appear to reduce inflammation‐induced premature senescence in the kidney.^37^ Further, systemic inflammation may contribute to portal hypertension and splanchnic vasodilation,^38^ leading to relative renal hypoperfusion. In this regard, we found that IL‐8 and TGF‐β2 were associated with incident AKI. These mechanisms could theoretically be mitigated by targeted immunomodulating therapies. Alternatively, steroids might counteract relative adrenal insufficiency, which is associated with AKI in decompensated cirrhosis^39^ and is readily treatable. Pentoxifylline treatment was not associated with a reduction in incident AKI, despite previous reports of a reduction in hepatorenal syndrome (HRS) in AH.^40^

Beta‐blockers, used to prevent portal hypertensive gastrointestinal bleeding, have been previously associated with AKI in AH.^41^ However, in patients with advanced cirrhosis without AH, the benefits of beta‐blockers appear to outweigh the risks^42^ and treatment with beta‐blockers has been associated with improved survival in patients with acute‐on‐chronic liver failure (ACLF).^43^ The association between AKI and beta‐blockers in AH has not yet been reproduced. Here, beta‐blocker use was not associated with AKI or D90 mortality. However, we cannot comment on beta‐blocker use in patients presenting with AH and severe infection, since patients with uncontrolled infection were excluded from recruitment to STOPAH.^1^

Infection and gastrointestinal bleeding at recruitment were not associated with D0 or incident AKI. However, an exclusion criterion for STOPAH was infection or gastrointestinal bleeding that could not be controlled within seven days.^1^ Incident infection at D7 associated with incident AKI but both associated with D90 mortality independent of one another. Patients with more severe disease appear to be at greater risk of both incidental infections^34^ and AKI.

We could not confirm the utility of a range of biomarkers previously linked to AKI in patients with cirrhosis.^11^ However, serum IL‐8 combined with bilirubin showed some promise as a potential D0 biomarker for incident AKI. Further, two miRNA species (miR‐6826‐5p and miR‐6811‐3p) may be potential biomarkers for incident AKI. However, these results must be interpreted with caution, as this analysis included a subset of participants. Interestingly, miR‐6826‐5p is an exosomal miRNA previously linked to liver injury in cholecystitis,^44^ and pancreatic and biliary tract cancers.^45^ Exosomal miRNAs are thought to be mediators of cell‐to‐cell communication.^46^ Less is known about miR‐6811‐3p, a vesicle miRNA linked to multiple sclerosis.^47^ These biomarkers require prospective validation in an independent cohort. Once confirmed, prospective trials of preventative therapy could be considered in ‘at risk’ patients – for example, prophylactic terlipressin, volume expansion, or consideration of immunomodulating therapy for kidney dysfunction.

Our data were derived from the largest prospectively recruited cohort of patients with AH to date. Although this study was not specifically designed to answer the question of AKI incidence and prediction, participants were prospectively recruited, well‐characterised, and most had serum available for biomarker exploration. Participants were recruited at multiple centres across the United Kingdom. As such, we are confident that our conclusions regarding the prevalence of AKI and the relationships between D0‐AKI, incident AKI and D90 mortality are robust.

However, our work has limitations. Firstly, patients with severe AKI (creatinine >500 μmol/L [>5.7 mg/dL] or RRT at screening) were excluded from STOPAH, so we likely underestimate the prevalence of D0‐AKI. Secondly, participants without D7 creatinine available because they died before D7 were considered ‘no AKI’ in the incident AKI analysis. This may introduce a degree of immortal time bias in the survival analyses and risk some misclassification in the regression analyses, as some may have developed an AKI before D7. Thirdly, participants alive at D7 without D7 creatinine available were excluded from this analysis – potentially excluding rapidly‐improving participants discharged before D7. It should be emphasised that we have taken a conservative approach to each of these limitations, which will have weakened the association of incident (D7) AKI with mortality. The sensitivity analyses demonstrated a stronger association with mortality when a more severe AKI classification (creatinine ≥133 μmol/L [1.5 mg/dL]) or a more favourable control classification (Figure 2iii) was used. We used the more conservative classifications to avoid type 1 errors and spurious associations, given the inherent limitations of retrospective analysis.

Similarly, there will have been participants with a creatinine above their baseline but less than 133 μmol/L who did not record a normal creatinine during follow‐up because they died. These participants will have been classified as non‐AKI, weakening the association between D0‐AKI and mortality. Thirty‐four participants had creatinine above 133 μmol/L and did not record a normal creatinine. These were classified as D0‐AKI but may actually have had chronic kidney disease. However, given the typical clinical characteristics of an AH population, it is more likely that they did not record a normal creatinine because they died within the first few weeks (twenty‐four of these participants had died by D28). Any bias from stable CKD misclassified as AKI is likely small.

Further, serum creatinine data were only available at the pre‐specified timepoints of the STOPAH protocol, viz. Days 0, 7, 14, 21, 28 and 90, precluding a daily assessment of the evolution of changes in serum creatinine. Specifically, we cannot be certain that increments in creatinine occurred within the 48‐hour period suggested by the ICA definition for AKI. Our methodology may have led to an increased categorisation of AKI for patients in whom the rise in serum creatinine occurred over a time course longer than 48 hours.

STOPAH was an interventional RCT of pharmacotherapy to reduce mortality in AH. Hence, detailed characterisation of the various types of AKI, from hepatorenal syndrome to acute tubular necrosis, was not possible. We do not have data on the prevalence of ascites. This may explain why certain biomarkers are not more closely associated with AKI; for example, NGAL is elevated primarily in acute tubular necrosis.^12^ Each type of AKI will have different effects on the cellular and molecular pathways in the kidney and may affect putative biomarkers differently.^48^ Certain biomarkers may have a role in distinguishing different types of AKI. Other biomarkers, for example IL‐18, may be more sensitive in the urine compared to the serum^12^; however, urine was not available. Finally, we used a fixed D7 cut‐off for incident AKI. This may explain why biomarkers such as cystatin C and B2M did not associate with incident AKI, as typically these only rise 24–48 h before serum creatinine.^12^, ^13^

These unanswered questions require prospective exploration, and we hope that our work describes the hitherto unrecognised need for such prospective exploration in patients with AH.

## 5 CONCLUSIONS

AKI at presentation (D0‐AKI) and incident AKI are associated with D90 mortality in AH. Incident AKI is associated with D90 mortality, independent of underlying liver disease severity. Incident AKI is associated with a higher mortality rate than AKI at presentation (D0‐AKI). Prednisolone treatment is associated with a reduced risk of developing incident AKI. Further work should focus on identifying patients most at risk of incident AKI, validating the biomarkers discovered, including novel miRNAs, and identifying treatments that can prevent incident AKI in those at risk.

## AUTHOR CONTRIBUTIONS

**Luke D. Tyson:** Conceptualization (equal); data curation (supporting); formal analysis (lead); investigation (lead); methodology (lead); project administration (lead); visualization (lead); writing – original draft (lead); writing – review and editing (lead). **Stephen Atkinson:** Conceptualization (equal); data curation (supporting); formal analysis (supporting); funding acquisition (supporting); investigation (supporting); methodology (supporting); supervision (supporting); writing – original draft (supporting); writing – review and editing (supporting). **Robert W. Hunter:** Data curation (supporting); formal analysis (supporting); investigation (supporting); methodology (supporting); visualization (supporting); writing – original draft (supporting); writing – review and editing (supporting). **Michael Allison:** Data curation (supporting); writing – review and editing (supporting). **Andrew Austin:** Data curation (supporting); writing – review and editing (supporting). **James Dear:** Data curation (supporting); formal analysis (supporting); investigation (supporting); methodology (supporting); resources (supporting); writing – review and editing (supporting). **Ewan Forrest:** Data curation (supporting); writing – review and editing (supporting). **Tong Liu:** Investigation (supporting); writing – review and editing (supporting). **Emma Lord:** Project administration (supporting); writing – review and editing (supporting). **Steven Masson:** Data curation (supporting); writing – review and editing (supporting). **Joao Nunes:** Investigation (supporting); writing – review and editing (supporting). **Paul Richardson:** Data curation (supporting); writing – review and editing (supporting). **Stephen D. Ryder:** Data curation (supporting); writing – review and editing (supporting). **Mark Wright:** Data curation (supporting); writing – review and editing (supporting). **Mark Thursz:** Conceptualization (equal); data curation (lead); funding acquisition (lead); resources (lead); supervision (supporting); writing – review and editing (supporting). **Nikhil Vergis:** Conceptualization (equal); data curation (supporting); formal analysis (supporting); investigation (supporting); methodology (supporting); project administration (supporting); supervision (lead); visualization (supporting); writing – original draft (supporting); writing – review and editing (supporting).

## FUNDING INFORMATION

This study was supported by the UK Medical Research Council (grant MR/R014019/1) and the National Institute for Health Research Imperial Biomedical Research Centre. STOPAH was supported by NIHR Health Technology Assessment Programme grant 08/14/44.

## CONFLICT OF INTEREST STATEMENT

NV was a fulltime employee of Imperial College London from October 2018 to March 2022 and has been a fulltime employee of GSK thereafter. Otherwise, the remaining authors (LDT, SA, RH, MA, AA, JWD, EF, TL, EL, SM, JN, PR, SDR, MW and MT) do not have any personal, professional or financial competing interests to disclose.

## PATIENT CONSENT STATEMENT

Written consent was obtained from each participant or from their legal representative until such time as they recovered mental capacity.

## PERMISSION TO REPRODUCE MATERIAL FROM OTHER SOURCES

Not applicable.

## CLINICAL TRIAL REGISTRATION

Not applicable; this is a secondary analysis of the STOPAH trial.

## Supporting information


Figure S1

Figure S2

Figure S3

Data S1
