# Supplementary figures and images for: In severe alcohol‐related hepatitis, acute kidney injury is prevalent, associated with mortality independent of liver disease severity, and can be predicted using IL‐8 and micro‐RNAs
Source: Aliment Pharmacol Ther. 2023 Oct 2;58(11-12):1217–29. doi: 10.1111/apt.17733 (PMC10946848; doi:10.1111/apt.17733)

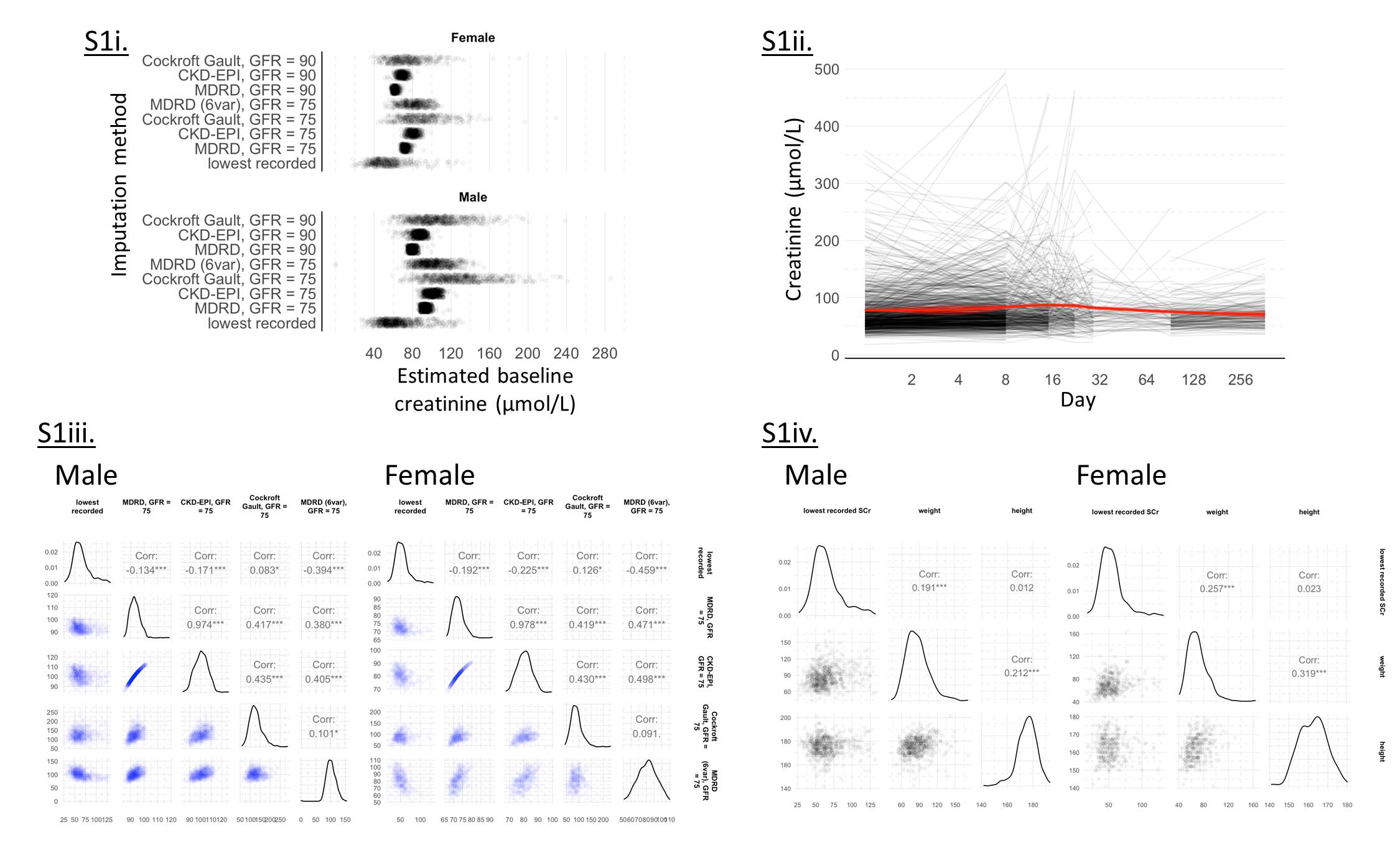

Supplement: Supplementary file 1 — Figure S1 [file APT-58-1217-s002.tif]

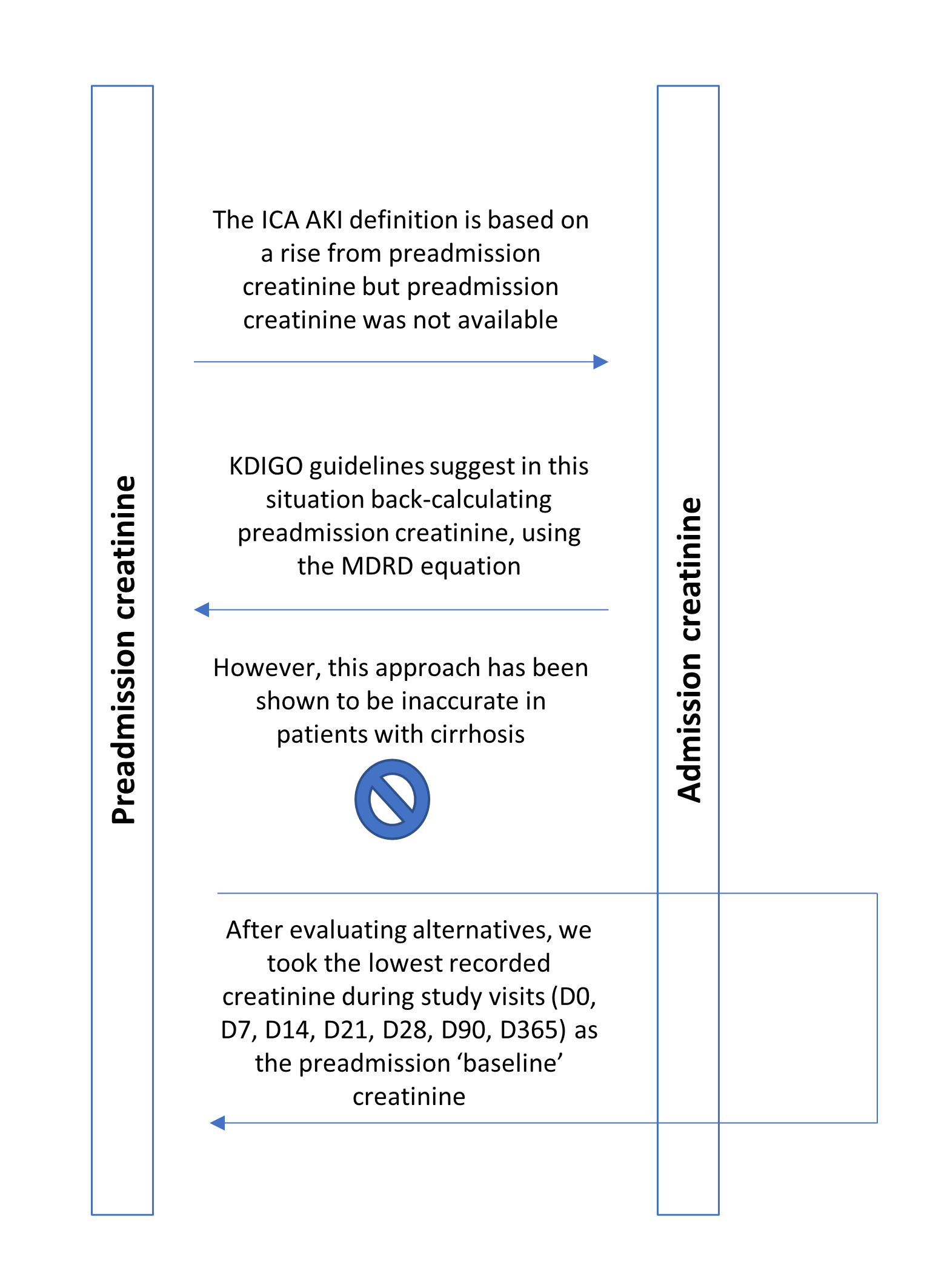

Supplement: Supplementary file 2 — Figure S2 [file APT-58-1217-s003.tif]

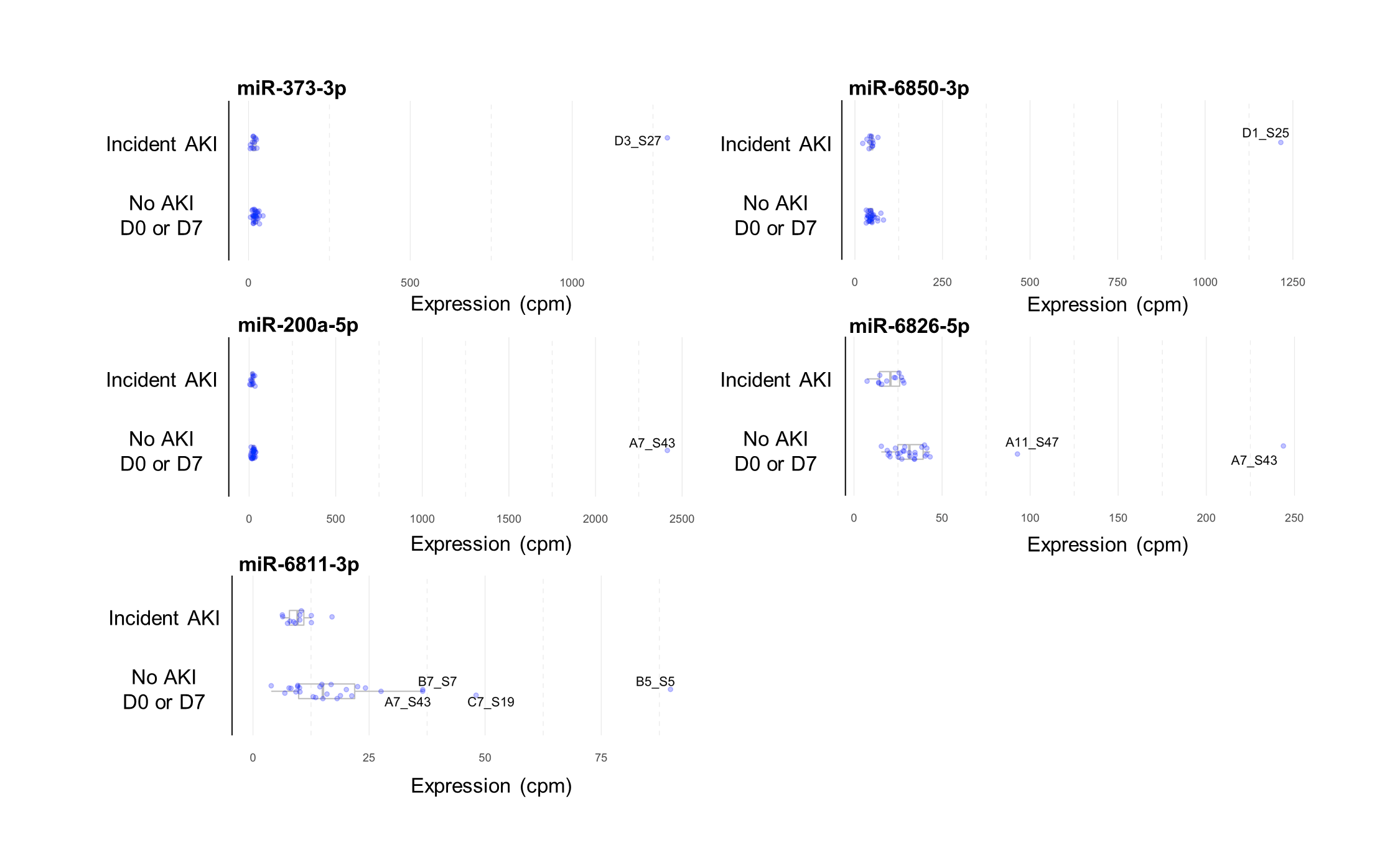

Supplement: Supplementary file 3 — Figure S3 [file APT-58-1217-s004.tif]
