# Supplementary material for: In severe alcohol‐related hepatitis, acute kidney injury is prevalent, associated with mortality independent of liver disease severity, and can be predicted using IL‐8 and micro‐RNAs
Source: Aliment Pharmacol Ther. 2023 Oct 2;58(11-12):1217–29. doi: 10.1111/apt.17733 (PMC10946848; doi:10.1111/apt.17733)
Supplement: Supplementary file 4 — Data S1 [file APT-58-1217-s001.docx]

**SUPPLEMENTARY METHODS**

**Steroids or pentoxifylline for alcoholic hepatitis trial inclusion criteria**

- Aged 18 years or older
- Clinical alcoholic hepatitis:
  - Serum bilirubin >80 μmol/L
  - History of excess alcohol (>80 g/day male, >60 g/day female) to within 2 months of randomisation
- Less than 4 weeks since admission to hospital
- Maddrey’s modified Discriminant function ≥32
- Informed consent

**Steroids or pentoxifylline for alcoholic hepatitis trial exclusion criteria**

- Abstinence of >2 months prior to randomisation
- Duration of clinically apparent jaundice >3 months
- Other causes of liver disease including:
- Evidence of chronic viral hepatitis
- Biliary obstruction
- Hepatocellular carcinoma
- Evidence of current malignancy (except non-melanotic skin cancer)
- Previous entry into the study, or use of either prednisolone or pentoxifylline within 6 weeks of admission
- AST >500 U/L or ALT >300 U/L
- Patients with a serum creatinine >500 μmol/L or requiring renal support
- Patients dependent upon inotropic support (adrenaline or noradrenaline; terlipressin allowed)
- Active gastrointestinal bleeding
- Untreated sepsis
- Patients with known hypersensitivity to pentoxifylline, other methyl xanthines, or any of the excipients
- Patients with cerebral haemorrhage, extensive retinal haemorrhage, acute myocardial infarction (within the last 6 weeks) or severe cardiac arrhythmias (not including atrial fibrillation)
- Pregnant or lactating women

**Total RNA isolation from serum using QIAGEN miRNease Serum/Plasma kit and RNA library preparation using the QIAseq microRNA Library kit**

Total RNA was isolated from 100 µL serum. Briefly, after ligation of 3’ and 5’ adapters, the samples underwent a cDNA synthesis step in which a unique molecular identifier (UMI) was assigned to each miRNA molecule. Samples were sequenced on a NextSeq 500/550 High Output Kit v2 (single-end 75 bp reads) and the output mapped to miRBase V21 using the QIAseq microRNA analysis pipeline. UMI counts were used to quantify unique miRNA molecules.

**SUPPLEMENTARY ANALYSIS**

**Comparison of lowest recorded to estimated baseline creatinine**

Historical serum creatinine data was not available for the STOPAH study cohort. We compared alternative methods for imputing a baseline serum creatinine concentration to the lowest recorded creatinine. Formula-based imputation methods over-estimated baseline creatinine relative to the lowest-recorded creatinine, even when a GFR of 90 ml/min was assumed (**Supplementary Figure 1i**). This is in keeping with the observation that patients with chronic liver disease have relatively low serum creatinine so eGFRs calculated using standard formulae significantly over-estimate true GFR, as discussed in **Methods**.

One potential concern with using the lowest recorded creatinine as a baseline is that it might be artefactually low in patients who have been admitted to hospital and subjected to iatrogenic haemodilution. If that were the case, one might expect this measure to show a nadir at 7 - 14 days after study enrolment. We did not observe any such nadir (**Supplementary Figure 1ii**), providing reassurance that the lowest-recorded creatinine is likely to give an undistorted measure of true baseline creatinine. The lowest-recorded serum creatinine correlated poorly with formula-based imputed baseline creatinine (**Supplementary Figure 1iii**) but correlated moderately well with body weight (**Supplementary Figure 1iv**). Therefore, of the methods available to us, the lowest-recorded serum creatinine is likely to give the most accurate estimate of true “baseline” serum creatinine.

**Sensitivity analyses complementary to main analyses described in manuscript: mortality**

Incident AKI remained associated with D90 mortality when adjusted for age and D0 MELD score (AHR 1.57, 1.13-2.20, P = 0.008), D0 bilirubin (AHR 1.88, 1.34-2.64, P <0.001), D0 INR (AHR 1.93, 1.39-2.67, P <0.001), age and D7 mDF (AHR 2.02, 1.35-3.02, P =0.001), D7 bilirubin (AHR 1.90, 1.30-2.77, P =0.001), and D7 INR (AOR 2.08, 1.31-3.30, P = 0.002).

**Sensitivity analyses complementary to main analyses described in manuscript: age**

Patient age was not associated with D0-AKI (OR per year 1.00, 0.99-1.00, P =0.568) or incident AKI (OR 1.01, 0.99-1.03, P =0.553), hence clinical associations and biomarkers were adjusted for mDF alone.

**Sensitivity analyses complementary to main analyses described in manuscript: clinical associations**

Gastrointestinal bleeding and infection at recruitment were not associated with D0-AKI (OR 0.76, 0.40-1.43, P =0.390; OR 1.20, 0.75-1.92, P =0.459, respectively). Hepatic encephalopathy at D0 (OR 1.14, 0.72-1.81, P =0.590), prior gastrointestinal bleeding (OR 1.25, 0.61-2.55, P =0.536), and infection at recruitment (OR 1.17, 0.64-2.13, P =0.609) were not associated with developing an incident AKI.

**Sensitivity analyses complementary to main analyses described in manuscript: prednisolone**

The association between prednisolone and incident AKI persisted when AKI was defined as creatinine ≥133 µmol/L (1.5 mg/dL): OR 0.49, 0.25-0.91, P =0.024; adjusted for D0-mDF AOR 0.50, 0.26-0.96, P =0.038.

Prednisolone treatment was not associated with D7 infection (OR 0.95, 0.64-1.39, P = 0.775) nor infection by D28 (OR 1.06, 0.79-1.41, P =0.701). However, prednisolone treatment was associated with infection developing between D28 and D90 (OR 1.66, 1.05-2.63, P =0.030), even when adjusted for mDF (AOR 1.66, 1.05-2.63, P =0.031) or MELD (AOR 1.65, 1.04-2.62, P =0.032). Hence, late infections may well have contributed to the increase in mortality in prednisolone treated participants between D28 and D90 – given developing an infection by D90 is strongly associated with mortality (HR 1.65, 1.29-2.10, P <0.001; AHR adjusted for age and mDF 1.35, 1.06-1.73, P =0.017).

**Sensitivity analyses complementary to main analyses described in manuscript: beta blockers**

Neither cardioselective beta-blockers (atenolol, bisoprolol, metoprolol or nebivolol) nor non-selective beta-blockers (propranolol, carvedilol, or nadolol) associated with D0-AKI (OR 1.64, 0.85-3.17, P =0.138; OR 0.92, 0.55-1.53, P =0.743, respectively). Specifically, neither carvedilol (n =24, OR 0.18, 0.03-1.37, P =0.098) nor propranolol (n =89, OR 1.10, 0.64-1.89, P =0.727) were associated with D0-AKI.

There was no association between cardioselective (OR 1.66, 0.62-4.42, P =0.309) or non-selective (OR 1.31, 0.71-2.40, P =0.386) beta blocker use, carvedilol (OR 0.58, 0.13-2.59, P =0.474) or propranolol (OR 1.59, 0.83-3.06, P =0.167) and incident AKI.

**SUPPLEMENTARY TABLES**

**Supplementary Table 1: Key demographic and clinical characteristics of the patient cohort for microRNA differential expression analysis**

*Abbreviations: AKI = acute kidney injury; D0 = day zero; D7 = day seven; D28 = day 28; D90 = day 90; INR = international normalised ratio; IQR = interquartile range; N = number; mDF = Maddey’s modifed Discriminant Function’ MELD = Model For End-Stage Liver Disease score*

|  | Incident (D7) AKI | Neither D0 nor incident AKI |
| --- | --- | --- |
| N (% of whole cohort) | 12 (31) | 27 (69) |
| Median age, years (IQR) | 48 (43-53) | 52 (42-60) |
| Male gender, N (% of group) | 9 (75) | 14 (52) |
| Death by D28, N (% of group) | 8 (67) | 13 (48) |
| Death by D90, N (% of group) | 9 (75) | 14 (52) |
| Beta-blocker use at presentation, N  (% of group) | 1 (8) | 2 (7) |
| Hepatic encephalopathy at presentation, N (% of group) | 5 (42) | 12 (44) |
| Gastrointestinal bleed at presentation, N (% of group) | 0 (0) | 0 (0) |
| Infection at presentation, N  (% of group) | 0 (0) | 1 (4) |
| Infection at D7, N (% of group) | 3 (25) | 6 (22) |
| Median D0 bilirubin, µmol/L (IQR) | 355 (229-445) | 322 (186-403) |
| Median D0 INR (IQR) | 2.0 (1.7-2.0) | 1.7 (1.5-1.9) |
| Median D0 creatinine, µmol/L (IQR) | 57 (46-73) | 64 (59-78) |
| Median mDF (IQR) | 65 (50-75) | 54 (41-76) |
| Median D0 MELD (IQR) | 29 (28-30) | 26 (23-29) |

**Supplementary Table 2: differences in the key demographic and clinical characteristics of the patient cohorts compared**

*Abbreviations and annotations: AKI = acute kidney injury; D0 = day zero; D7 = day seven; D28 = day 28; D90 = day 90; INR = international normalised ratio; IQR = interquartile range; N = number; mDF = Maddey’s modifed Discriminant Function’ MELD = Model For End-Stage Liver Disease score.*

^†^*Continuous parametric variables were compared by Student’s t-test, non-parametric by Mann-Whitney U test, and categorical by χ2 test*

|  | D0 AKI | No D0 AKI | *P-value*^†^ | Incident (D7)  AKI | Neither D0 nor incident AKI | *P-value*^†^ |
| --- | --- | --- | --- | --- | --- | --- |
| N  (% of whole cohort) | 198 (19) | 853 (81) | *NA* | 119 (11) | 452 (43) | *NA* |
| Median age, years  (IQR) | 50 (42-56) | 49 (42-56) | 0.601 | 49 (43-57) | 49 (42-57) | 0.519 |
| Male gender, N  (% of group) | 124 (63) | 537 (63) | 0.931 | 77 (65) | 273 (60) | 0.391 |
| Death by D28, N  (% of group) | 44 (22) | 122 (14) | **0.006** | 45 (38) | 65 (14) | **<0.001** |
| Death by D90, N  (% of group) | 63 (32) | 210 (25) | **0.037** | 56 (47) | 115 (25) | **<0.001** |
| Prednisolone therapy, N (% of group) | 113 (57) | 412 (48) | **0.026** | 39 (33) | 213 (47) | **0.005** |
| Pentoxifylline therapy, N (% of group) | 105 (53) | 422 (49) | 0.367 | 59 (50) | 216 (48) | 0.728 |
| Beta-blocker use at D0, N (% of group) | 27 (14) | 122 (14) | 0.809 | 22 (18) | 57 (13) | 0.099 |
| Hepatic encephalopathy at presentation, N  (% of group) | 70 (35) | 187 (22) | **<0.001** | 31 (26) | 107 (24) | 0.590 |
| Gastrointestinal bleed at presentation, N  (% of group) | 12 (6) | 67 (8) | 0.388 | 11 (9) | 34 (8) | 0.535 |
| Infection at presentation, N (% of group) | 25 (13) | 92 (11) | 0.458 | 16 (13) | 53 (12) | 0.609 |
| Infection at D7, N  (% of group) | 31 (16) | 84 (10) | **0.019** | 23 (19) | 48 (11) | **0.010** |
| Median D0 bilirubin, µmol/L (IQR) | 386  (247-520) | 243  (157-387) | **<0.001** | 390  (262-470) | 260  (177-385) | **<0.001** |
| Median D0 INR (IQR) | 1.8 (1.6-2.2) | 1.8 (1.5-2.0) | 0.057 | 1.8 (1.6-2.2) | 1.7 (1.5-2.0) | **0.004** |
| Median D0 creatinine, µmol/L (IQR) | 123 (91-181) | 61 (51-73) | **<0.001** | 66 (52-82) | 60 (50-72) | **0.003** |
| Median mDF (IQR) | 63 (49-89) | 54 (43-71) | **<0.001** | 64 (52-89) | 53 (43-70) | **<0.001** |
| Median D0 MELD (IQR) | 30 (26-34) | 25 (22-28) | **<0.001** | 28 (25-30) | 25 (23-28) | **<0.001** |

**Supplementary Table 3: acute kidney injury stage at day zero and day seven with corresponding 90-day mortality**

*Abbreviations and annotations: AKI = acute kidney injury; D0 = day zero; D7 = day seven; N = number*

|  | International Club of Ascites AKI stage | | | | |
| --- | --- | --- | --- | --- | --- |
|  | 0 (no AKI) | 1 | 2 | 3 | Unable to classify |
| AKI stage at D0, N (%) | 853 | 114 | 37 | 16 | 31 |
| D90 mortality stratified  for D0 AKI stage, N (%) | 210 (25) | 25 (22) | 12 (32) | 3 (19) | 23 (74) |
| Spearman's *rho*,  ordinal by ordinal (P value) | -0.004 (0.901) | | | | *Excluded* |
| AKI stage at D7, N (%) | 452 | 77 | 31 | 11 | 0 |
| D90 mortality stratified  for D7 AKI stage, N (%) | 115 (25) | 32 (42) | 17 (55) | 7 (64) | 0 (0) |
| Spearman's *rho*,  ordinal by ordinal (P value) | 0.199 (**<0.001**) | | | | *N/A* |

**Supplementary Table 4: differences in clinical and novel biomarkers between participants with and without day zero AKI in the prednisolone and pentoxifylline-treated groups**

*Abbreviations and annotations: AKI = acute kidney injury; D0 = day zero; INR = international normalised ratio; IQR = interquartile range*

^†^*Continuous non-parametric variables were compared by Mann-Whitney U test*

|  | Prednisolone treated | | | Pentoxifylline treated | | |
| --- | --- | --- | --- | --- | --- | --- |
|  | D0 AKI | No D0 AKI | P-value† | D0 AKI | No D0 AKI | P-value† |
| Median D0 white blood cell count, x10^9^ (IQR) | 11.3 (7.9-15.1) | 8.3 (6.1-11.6) | **<0.001** | 10.4 (7.9-14.9) | 8.2 (5.9-12.1) | **<0.001** |
| Median D0 bilirubin, µmol/L (IQR) | 402 (255-522) | 243 (151-376) | **<0.001** | 414 (291-532) | 238 (156-382) | **<0.001** |
| Median D0 INR (IQR) | 1.8 (1.6-2.1) | 1.8 (1.5-2.0) | 0.224 | 1.8 (1.6-2.2) | 1.8 (1.6-2.0) | 0.460 |
| Median D0 sodium, mEq/L (IQR) | 134 (128-136) | 134 (131-137) | 0.062 | 133 (128-136) | 134 (131-137) | **0.033** |
| Median D0 albumin, g/L (IQR) | 26 (20-30) | 24 (21-28) | 0.645 | 26 (21-30) | 25 (22-28) | 0.409 |
| Median D0 cystatin C, mg/L (IQR) | 1.50 (0.75-3.75) | 1.00 (0.65-2.07) | **0.010** | 1.50 (0.89-3.22) | 1.16 (0.69-2.24) | **0.048** |
| Median D0 NGAL, ng/mL (IQR) | 466 (253-1080) | 349 (143-769) | **0.014** | 458 (254-811) | 349 (170-838) | 0.179 |
| Median D0 beta-2 microglobulin (µg/mL) | 0.20 (0.07-1.13) | 0.10 (0.04-0.27) | **<0.001** | 0.27 (0.07-1.09) | 0.10 (0.04-0.28) | **<0.001** |
| Median D0 IL-6, pg/mL (IQR) | 29.7 (12.7-47.9) | 17.9 (11.3-30.0) | **<0.001** | 26.0 (17.4-41.3) | 18.2 (12.4-28.9) | **<0.001** |
| Median D0 IL-8, ng/mL (IQR) | 0.47 (0.20-0.71) | 0.39 (0.18-0.81) | 0.732 | 0.51 (0.25-0.89) | 0.44 (0.20-0.86) | 0.458 |
| Median D0 IL-18, ng/mL (IQR) | 1.18 (0.69-2.04) | 0.97 (0.58-1.41) | **0.016** | 1.03 (0.67-1.71) | 0.98 (0.65-1.45) | 0.236 |
| Median D0 IL-22, pg/mL (IQR) | 4.85 (2.09-13.05) | 2.27 (0.84-5.84) | **<0.001** | 2.56 (1.25-5.87) | 2.20 (0.92-5.21) | 0.142 |
| Median D0 TGF-β1, ng/mL (IQR) | 5.35 (2.99-11.27) | 4.39 (1.81-9.55) | 0.139 | 5.70 (2.93-9.87) | 4.28 (1.80-9.57) | 0.056 |
| Median D0 TGF-β2, ng/mL (IQR) | 0.20 (0.10-0.35) | 0.14 (0.07-0.28) | **0.007** | 0.19 (0.10-0.37) | 0.14 (0.07-0.28) | **0.005** |
| Median D0 TNF-α, pg/mL (IQR) | 4.70 (3.33-7.04) | 3.90 (2.74-5.49) | **0.010** | 4.69 (3.35-6.05) | 3.81 (2.81-5.43) | **0.016** |

**Supplementary Table 5: differences in clinical and novel biomarkers between participants with and without incident (day seven) AKI in the prednisolone and pentoxifylline-treated groups**

*Abbreviations and annotations: AKI = acute kidney injury; D0 = day zero; D7 = day seven; INR = international normalised ratio; IQR = interquartile range*

^†^*Continuous non-parametric variables were compared by Mann-Whitney U test*

|  | Prednisolone treated | | | Pentoxifylline treated | | |
| --- | --- | --- | --- | --- | --- | --- |
|  | D7 AKI | No D7 AKI | P-value† | D7 AKI | No D7 AKI | P-value† |
| Median D0 white blood cell count, x10^9^ (IQR) | 9.7 (6.7-15.2) | 8.6 (6.3-11.9) | 0.125 | 8.8 (6.6-12.8) | 8.4 (5.9-12.2) | 0.508 |
| Median D0 bilirubin, µmol/L (IQR) | 377 (238-493) | 273 (177-407) | **0.003** | 377 (237-475) | 258 (175-392) | **0.001** |
| Median D0 INR (IQR) | 1.9 (1.7-2.3) | 1.7 (1.5-2) | **0.019** | 2.0 (1.8-2.4) | 1.7 (1.6-2.0) | **<0.001** |
| Median D0 sodium, mEq/L (IQR) | 134 (132-136) | 134 (131-137) | 0.724 | 134 (131-137) | 134 (130-137) | 0.985 |
| Median D0 albumin, g/L (IQR) | 23 (20-26) | 25 (21-28) | 0.072 | 23 (21-26) | 25 (21-28) | 0.135 |
| Median D0 cystatin C, mg/L (IQR) | 0.81 (0.55-2.07) | 0.92 (0.58-1.93) | 0.521 | 0.98 (0.68-2.39) | 1.00 (0.61-1.96) | 0.432 |
| Median D0 NGAL, ng/mL (IQR) | 232 (97-1260) | 248 (112-659) | 0.957 | 352 (236-1079) | 299 (147-784) | 0.163 |
| Median D0 beta-2 microglobulin (µg/mL) | 0.13 (0.04-0.28) | 0.10 (0.04-0.34) | 0.886 | 0.23 (0.06-0.40) | 0.09 (0.03-0.27) | **0.022** |
| Median D0 IL-6, pg/mL (IQR) | 27.5 (14.7-43.9) | 19.3 (12.7-33.4) | 0.165 | 23.1 (15.8-35.0) | 18.4 (13.2-31.2) | 0.100 |
| Median D0 IL-8, ng/mL (IQR) | 0.47 (0.18-1.25) | 0.39 (0.18-0.83) | 0.343 | 0.64 (0.24-1.38) | 0.43 (0.20-0.88) | 0.096 |
| Median D0 IL-18, ng/mL (IQR) | 0.89 (0.49-1.22) | 1.04 (0.52-1.42) | 0.489 | 0.89 (0.63-1.46) | 0.93 (0.60-1.41) | 0.811 |
| Median D0 IL-22, pg/mL (IQR) | 2.72 (1.57-6.40) | 2.55 (0.78-6.07) | 0.485 | 3.04 (1.16-8.11) | 2.49 (1.13-5.38) | 0.361 |
| Median D0 TGF-β1, ng/mL (IQR) | 7.00 (1.84-10.28) | 4.72 (2.12-10.23) | 0.920 | 4.30 (1.91-10.62) | 4.46 (2.06-8.88) | 0.549 |
| Median D0 TGF-β2, ng/mL (IQR) | 0.22 (0.08-0.36) | 0.15 (0.08-0.28) | 0.141 | 0.17 (0.11-0.33) | 0.14 (0.08-0.27) | 0.105 |
| Median D0 TNF-α, pg/mL (IQR) | 4.56 (2.00-8.55) | 3.97 (3.01-5.88) | 0.789 | 4.18 (2.69-7.77) | 3.62 (2.75-4.99) | 0.176 |

**Supplementary Table 6: top five most differentially expressed miRNAs**

*Abbreviations: logFC = log_2_fold-change; logCPM = log_2_counts per million; FDR = false-discovery rate (by Benjamini-Hochberg method)*

| Micro RNA | logFC | logCPM | F | P value | FDR |
| --- | --- | --- | --- | --- | --- |
| miR-373-3p | 2.57 | 5.58 | 32.6 | 0.0000004 | 0.0008478 |
| miR-6850-3p | 1.60 | 6.33 | 19.8 | 0.0000413 | 0.0389590 |
| miR-200a-5p | -2.52 | 6.15 | 12.0 | 0.0010413 | 0.5598217 |
| miR-6826-5p | -0.99 | 5.23 | 11.7 | 0.0011880 | 0.5598217 |
| miR-6811-3p | -1.04 | 4.38 | 11.0 | 0.0015725 | 0.5928345 |
